# The Proinsulin C-peptide—A Multirole Model

**DOI:** 10.1080/15438600490424389

**Published:** 2004

**Authors:** Donald F. Steiner

**Affiliations:** 1 Howard Hughes Medical Institute and Department of Biochemistry and Molecular Biology University of Chicago Chicago Illinois 60637 USA

## Abstract

The C-peptide links the insulin A and B chains in proinsulin, providing thereby a means to promote their efficient folding and assembly in the endoplasmic reticulum during insulin biosynthesis. It then facilitates the intracellular transport, sorting, and proteolytic processing of proinsulin into biologically active insulin in the maturing secretory granules of the *β* cells. These manifold functions impose significant constraints on the C-peptide structure that are conserved in evolution. After cleavage of proinsulin, the intact C-peptide is stored with insulin in the soluble phase of the secretory granules and is subsequently released in equimolar amounts with insulin, providing a useful independent indicator of insulin secretion. This brief review highlights many aspects of its roles in biosynthesis, as a prelude to consideration of its possible additional role(s) as a physiologically active peptide after its release with insulin into the circulation in vivo.


*Experimental Diab. Res.*, 5:7–14, 2004
Copyright © Taylor and Francis Inc.
ISSN: 1543-8600 print / 1543-8619 online
DOI: 10.1080/15438600490424389

Taylor & Francis
Taylor & Francis Group

### The Proinsulin C-peptide—A Multirole Model

**Donald F. Steiner**

*Howard Hughes Medical Institute and Department of Biochemistry and Molecular Biology, University of Chicago, Chicago, Illinois, USA*

**The C-peptide links the insulin A and B chains in proinsulin, providing thereby a means to promote their efficient folding and assembly in the endoplasmic reticulum during insulin biosynthesis. It then facilitates the intracellular transport, sorting, and proteolytic processing of proinsulin into biologically active insulin in the maturing secretory granules of the β cells. These manifold functions impose significant constraints on the C-peptide structure that are conserved in evolution. After cleavage of proinsulin, the intact C-peptide is stored with insulin in the soluble phase of the secretory granules and is subsequently released in equimolar amounts with insulin, providing a useful independent indicator of insulin secretion. This brief review highlights many aspects of its roles in biosynthesis, as a prelude to consideration of its possible additional role(s) as a physiologically active peptide after its release with insulin into the circulation in vivo.**

**Keywords** Beta Cell; Evolution of Function; IGF 1 and 2; Insulin Biosynthesis; Prohormone Convertases; Protein Folding; Secretory Pathway

Received 2 July 2003; accepted 18 October 2003.

Many individuals have contributed to the development of our basic knowledge of the proinsulin C-peptide over the last 35 years, including Jeffrey Clark, Arthur Rubenstein, Philip Oyer, Franco Melani, James D. Peterson, Susan Terris, Wolfgang Kemmler, Judith King, Sooja Cho Nehrlich, Howard Tager, Simon Kwok, Shu Jin Chan, Kenneth Polansky, Graeme Bell, Que-Ping Cao, Chris Nolan, Emmanual Margoliash, Michael Weiss, Jan Markussen (Novo Nordisk), Ronald Chance, and Bruce Frank (Eli Lilly and Co.). The author also wishes to thank Rosie Ricks for expert assistance in preparing this manuscript. Work from the author's laboratory has been supported by grants from the National Institutes of Health (DK20595 and DK13914) and by the Howard Hughes Medical Institute.

Address correspondence to Donald F. Steiner, Howard Hughes Medical Institute, The University of Chicago, Chicago, IL 60637, USA.

### INTRODUCTION

Intracellular cleavage of protein and polypeptide precursors is now recognized as a widely occurring biosynthetic mechanism that can play many important roles in preparing biologically important molecules for their function. Proinsulin was the first precursor protein of this type to be identified, sequenced, and shown to precede insulin in appropriately designed pulse-chase biosynthetic labeling experiments (Steiner et al., 1969). In insulin biosynthesis, it fulfills the important biologic role of facilitating the formation of the correct secondary and tertiary structure of the hormone. Once this function has been accomplished at the level of the rough endoplasmic reticulum (RER), newly formed proinsulin moves within minutes to the Golgi region of the β cell, where its proteolytic processing to insulin and C-peptide is initiated. The sequestration of this process within the *trans*-Golgi network (TGN) and immature secretory granules results in the retention of both the C-peptide and insulin in the storage granules in equal amounts, and both peptides are then released together into the circulation during active secretion (exocytosis) (Steiner et al., 2000a). These are the two major secreted products of the β cell, but the secretory granules also contain a variety of other peptides, including small amounts of proinsulin and some intermediates of proinsulin processing, islet amyloid polypeptide (IAPP, aka amylin), chromogranins, and many other minor components of possible functional significance (Nishi et al., 1990).

### THE C-PEPTIDE RIA AND ITS CLINICAL APPLICATION

Because of the secretion of insulin and C-peptide in nearly equimolar amounts, measurements of immunoreactive C-peptide release has proven to be highly useful as an independent measure of insulin secretory rate in vivo in humans,

| | 1 2 3 4 5 6 7 8 9 10 11 12 13 14 15 16 17 18 19 20 21 22 23 24 25 26 27 28 29 30 31 32 33 34 35 36 37 38 |
|---|---|
| HUMAN | Glu-Ala-Glu-Asp-Leu-Gln-Val-Gly-Gln-Val-Glu-Leu-Gly-Gly-Gly-Pro-Gly-Ala-Gly-Ser-Leu-Gln-Pro-Leu-Ala-Leu-Glu-Gly-Ser-Leu-Gln |
| MONKEY | Glu-Ala-Glu-Asp-Pro-Gln-Val-Gly-Gln-Val-Glu-Leu-Gly-Gly-Gly-Pro-Gly-Ala-Gly-Ser-Leu-Gln-Pro-Leu-Ala-Leu-Glu-Gly-Ser-Leu-Gln |
| HORSE | Glu-Ala-Glu-Asp-Pro-Gln-Val-Gly-Glu-Val-Glu-Leu-Gly-Gly-Gly-Pro-Gly-Leu-Gly-Gly-Leu-Gln-Pro-Leu-Ala-Leu-Ala-Gly-Pro-Gln-Gln |
| PIG | Glu-Ala-Glu-Asn-Pro-Gln-Ala-Gly-Ala-Val-Glu-Leu-Gly-Gly-Gly-Leu-Gly — Gly — Leu-Gln-Ala-Leu-Ala-Leu-Glu-Gly-Pro-Pro-Gln |
| COW, LAMB | Glu-Val-Glu-Gly-Pro-Gln-Val-Gly-Ala-Leu-Glu-Leu-Ala-Gly-Gly-Pro-Gly-Ala-Gly-Gly-Leu — — — — — Glu-Gly-Pro-Pro-Gln |
| RABBIT | Glu-Val-Glu-Glu-Leu-Gln-Val-Gly-Gln-Ala-Glu-Leu-Gly-Gly-Gly-Pro-Gly-Ala-Gly-Gly-Leu-Gln-Pro-Ser-Ala-Leu-Glu — Ala-Leu-Gln |
| DOG | Glu-Val-Glu-Asp-Leu-Gln-Val-Arg-Asp-Val-Glu-Leu-Ala-Gly-Ala-Pro-Gly-Glu-Gly-Gly-Leu-Gln-Pro-Leu-Ala-Leu-Glu-Gly-Ala-Leu-Gln |
| RAT I | Glu-Val-Glu-Asp-Pro-Gln-Val-Pro-Gln-Leu-Glu-Leu-Gly-Gly-Gly-Pro-Glu-Ala-Gly-Asp-Leu-Gln-Thr-Leu-Ala-Leu-Glu-Val-Ala-Arg-Gln |
| RAT II | Glu-Val-Glu-Asp-Pro-Gln-Val-Ala-Gln-Leu-Glu-Leu-Gly-Gly-Gly-Pro-Gly-Ala-Gly-Asp-Leu-Gln-Thr-Leu-Ala-Leu-Glu-Val-Ala-Arg-Gln |
| GUINEA PIG | Glu-Leu-Glu-Asp-Pro-Gln-Val-Glu-Gln-Thr-Glu-Leu-Gly-Met-Gly-Leu-Gly-Ala-Gly-Gly-Leu-Gln-Pro-Leu-Ala-Leu-Glu-Met-Ala-Leu-Gln |
| CHINCHILLA | Glu-Leu-Glu-Asp-Pro-Gln-Val-Gly-Gln-Ala-Asp-Pro-Gly-Val-Val-Pro-Glu-Ala-Gly-Arg-Leu-Gln-Pro-Leu-Ala-Leu-Glu-Met-Thr-Leu-Gln |
| DUCK | Asp-Val-Glu-Gln-Pro-Leu-Val-Asn-Gly-Pro — Leu-His-Gly-Glu-Val-Gly-Glu — — Leu-Pro-Phe-Gln-His-Glu-Glu — — Tyr-Gln |
| CHICKEN | Asp-Val-Glu-Gln-Pro-Leu-Val-Ser-Ser-Pro — Leu-Arg-Gly-Glu-Ala-Gly-Val — — Leu-Pro-Phe-Gln-Gln-Glu-Glu-Tyr-Glu-Lys-Val |
| ANGLERFISH | Asp-Val-Asp-Gln-Leu-Leu-Gly-Phe-Leu-Pro-Pro-Lys-Ser-Gly-Gly-Ala-Ala-Ala-Ala-Gly-Ala-Asp-Asn-Glu-Val-Ala-Glu-Phe-Ala-Phe-Lys-Asp-Gln-Met-Glu-Met-Met-Val |
| HAGFISH | Asp-Thr-Gly-Ala-Leu-Ala-Ala-Phe-Leu-Pro-Leu-Ala-Tyr-Ala-Glu-Asp-Asn-Glu-Ser-Gln-Asp-Asp-Glu-Ser-Ile-Gly-Ile-Asn-Glu-Val-Leu-Lys-Ser |
| AMPHIOXUS | Ser-Val-Ser-Lys-Arg-Ala-Ile-Asp-Phe-Ile-Ser-Glu-Gln-Gln-Ala-Lys-Asp-Tyr-Met-Gly-Ala-Met-Pro-His-Ile |
| MOLLUSC | Asn-Ala-Glu-Thr-Asp-Leu-Asp-Asp-Pro-Leu-Arg-Asn-Ile-Lys-Leu-Ser-Ser-Glu-Ser-Ala-Leu-Thr-Tyr-Leu-Thr |

**FIGURE 1**
Amino acid sequences of proinsulin C-peptides from selected vertebrates and a mollusc, *Lymnae stagnalis* (for sources see Steiner, 1984; Smit, 1996; Chan, 1990).

especially in diabetic subjects receiving exogenous insulin injections (Rubenstein et al., 1969; Polonsky et al., 1986). Although some intragranular processing of rat C-peptide has been reported (Verchere et al., 1996), the C-peptide in man does not undergo significant further processing prior to its release and also is quite stable in the circulation, exhibiting a very slow turnover rate (>30 minutes) (Polonsky et al., 1986; Verchere et al., 1996). This metabolic behavior contrasts with the rapid removal of insulin from the circulation via insulin receptor–mediated uptake followed by lysosomal degradation (Steiner et al., 2000b; Polonsky and O'Meara, 2001). The half-life of insulin is therefore much shorter than that of C-peptide—approximately 3.0 minutes or less under normal conditions. These large differences in metabolic clearance must be taken into account in translating C-peptide values into insulin secretory rates in vivo. Excellent methods for deconvolution of C-peptide values have been developed by Polonsky and O'Meara (2001) and have enabled the widespread use of serum/plasma C-peptide assays for evaluating $\beta$-cell reserve in both diabetic and normal subjects.

### C-PEPTIDE STRUCTURE

The mammalian proinsulin C-peptide is typically 31 amino acids in length and contains 4 or 5 acidic residues and, in only a few species, a single basic residue (Figure 1). It is highly variable in structure, but generally is devoid of aromatic amino acids. Although many studies have failed to detect ordered structures in the C-peptide (Weiss et al., 1990), more recent equilibrium denaturation studies have indicated that it is not a random coil, but rather contains detectable ordered structure both when free or attached to insulin in proinsulin (Brems et al., 1990). Moreover, the presence of the C-peptide in proinsulin influences insulin core packing evidently through the formation of some kind of local stable structure at the C-A junction (Weiss et al., 1990). The central region of the C-peptide usually contains a glycine-rich segment that would be expected to confer great flexibility, allowing it to bend back upon itself to form a U-shaped or hairpin structure when attached to the insulin chains in native proinsulin where the C-terminus of the B chain domain and the N-terminus of the A chain domain are only 8 to 10 Å apart, based on x-ray analysis of crystalline insulin (Blundell et al., 1972). The shortest known mammalian C-peptide is 26 amino acids in length, as in bovine proinsulin (Nolan et al., 1971). A length of about 30 residues is a highly conserved feature even in the proinsulin-like peptides found in many invertebrates (including molluscs and arthropods) (Smit et al., 1996; Lagueux et al., 1990; Kondo et al., 1996), but not in nematodes such as *Caenorhabditis elegans* (Duret et al., 1998). Earlier C-peptide isolates from dog pancreas consisted of a

23–amino acid peptide (Peterson et al., 1972), but this turned out to be only a fragment of a typical 31–amino acid C-peptide that is encoded in the dog insulin gene (Kwok et al., 1983). The reason for this is that the dog C-peptide contains one of those rarely occurring basic residues, an arginine at position 8 (Figure 1), which evidently is processed by one of the prohormone convertases during the conversion of dog proinsulin to insulin. This results in storage of the N-terminally truncated 23-residue C-peptide in the pancreatic islets. Whether the N-terminal octapeptide is also stored and released with the other products during secretion has not been examined, but seems quite likely to be the case. However, it would probably lack the C-terminal arginine moiety (see below).

### MECHANISM OF PROCESSING OF PROINSULIN

The conversion of proinsulin to insulin, like the processing of many other neuroendocrine precursors, occurs through the combined action of the prohormone convertases PC2 and PC1/3. These are serine endoproteases having a high degree of specificity for cleavage after paired basic amino acid sequences, such as Lys-Arg or Arg-Arg (Zhou et al., 1999; Muller and Lindberg, 1999; Seidah and Chrétien, 1999). Their action is followed by that of carboxypeptidase-E (CPE), a carboxypeptidase-B–like enzyme having a high specificity for C-terminal basic residues (Fricker et al., 1986). The latter enzyme(s) removes the C-terminal basic amino acids exposed by the endoproteases, resulting in the generation of native insulin and C-peptide free of any linking basic residues. Without CPE, the buildup of such extended intermediate products (insulin-Arg-Arg or C-peptide-Lys-Arg) inhibits the action of the convertases, resulting in incomplete processing of precursors (Day et al., 1998). Disruption or inactivating mutations of the genes encoding PC2, PC1/3, or CPE all lead to marked defects in proinsulin processing in mice (Furuta et al., 1998; Zhu et al., 2002; Naggert et al., 1995).

Under normal conditions, the processing of proinsulin in the secretory granules is highly efficient, yielding over 95% insulin and C-peptide, with only small amounts of residual proinsulin and intermediates (Steiner et al., 2000a). However, in the presence of $\beta$-cell stress, as in untreated type 1 or type 2 diabetes, processing of proinsulin may be less complete and this defect is then magnified in the circulation due to the significantly slower clearance of proinsulin (resulting mainly from its reduced receptor affinity), sometimes leading to highly elevated circulating proinsulin levels. The basis of this defect in the diabetic remains unclear, but probably arises from a combination of factors, including impaired granule retention and/or acidification and reduced expression of convertases (Rhodes and Alarcón, 1994).

### EVOLUTIONARY ASPECTS OF INSULIN AND C-PEPTIDE

Although it is now clear that 2-chain insulin molecules are found very widely in the animal kingdom, other insulin-related proteins also exist in many species. Among these are the two closely related insulin-like growth factors, IGFs 1 and 2. These extended single-chain molecules have been found so far only in vertebrates (LeRoith and Roberts, 1993). They share similarities both in structure and action to insulin, exerting their effects via the homologous IGF1 receptor, which is also a heterotetrameric tyrosine kinase family member like the insulin receptor (De Meyts and Whittaker, 2002). Their divergence from a common insulin-like ancestor early in the course of vertebrate evolution (Chan et al., 1990) appears to have assisted the partition of growth regulation (IGFs) from metabolic regulation (insulin), although with some functional overlap due to the mutual interdependence of these processes and also a low level of receptor cross-talk. In invertebrates, classic insulin-like peptides often regulate both activities or have a more prominent role in growth control (Brogiolo et al., 2001).

The uncleaved integral C-domains in IGFs 1 and 2 are considerably shorter than the C-peptide in proinsulin and also appear to contribute to receptor binding (Cara et al., 1990). The A chain domains are also extended variably through a short D domain and a longer E domain. The latter domain is removed during processing of the IGFs by furin and/or other members of the prohormone convertase family during their constitutive secretion from a wide variety of cells in the body (Duguay et al., 1997, 1998). The shorter C domain in these insulin-like forms suggests that overall length in the proinsulin C-peptides may be a required feature for successful processing of proinsulin to insulin rather than for folding per se (see below). A variety of other studies have shown that shortened connecting segments do not prevent proinsulin from folding properly and forming the correct disulfide bonds of insulin. Indeed, it has been suggested that directly linked single-chain B-A constructs completely devoid of a connecting peptide can fold and be normally secreted by neuroendocrine cells (Powell et al., 1988). However, more recent studies have shown that such molecules have a strong tendency to mispair disulfide bridges during folding (Liu et al., 2003). They also are almost devoid of biological activity unless cleaved (Derewenda et al., 1991). A minimal C-peptide chain length of several amino acids thus seems to be an essential requirement for successful biosynthesis of the prohormone. Even IGF1 with its shortened C domain of 11 residues tends to partially interchange the disulfide bridges involving the 2 adjacent cysteine residues at positions A6 and A7 under some conditions, but this misfolding event appears to be due mainly to differences between the insulin and IGF B chain sequences (Hua et al., 2002).

Although the IGFs have a shorter than normal C domain, the connecting peptide segment of prorelaxin, which gives rise to relaxin, a more distant relative of insulin with a similar 2-chain structure, is 104 residues in length in humans (Hudson et al., 1984). Little is known regarding the proteolytic processing of prorelaxin or its products, although it also appears likely to involve members of the subtilisin-like prohormone convertase family. The much greater length of the prorelaxin "C-peptide" suggests the possibility that additional biologically active sequences may reside therein. Alternatively, it could merely reflect (more faithfully than does proinsulin) the structure of an ancestral insulin-like gene that might have arisen via exon shuffling within a considerably larger protein (Steiner 1984). In any case, the existence of prorelaxin, as well as 2 additional mammalian insulin-like peptides (Koman et al., 1996; Burkhardt et al., 1994) shows that a much larger connecting peptide segment than seen in proinsulin can also support correct folding of an insulin-like structure and this has also been confirmed experimentally by doubling the C-peptide in an expressed construct (Wei et al., 1995).

### THE ROLES OF THE C-PEPTIDE IN INSULIN BIOSYNTHESIS

Although the connecting segment clearly plays a key role in insulin biosynthesis by ensuring the efficient and correct pairing of the A and B chains during the folding of proinsulin within the cysternae of the endoplasmic reticulum as discussed above, it also has a large number of additional constraints on its structure other than mere length. Mutations at selected sites in the C-peptide in human proinsulin have been shown to influence the refolding properties of the prohormone (Chen et al., 2002). However, because it mainly functions as a flexible connector to convert insulin chain combination into a unimolecular, rather than an otherwise bimolecular process, its structure also reflects this role. Many of these structural constraints are listed in Table 1 and will be briefly considered below. Clearly, the C-peptide must be removed from proinsulin to optimize the biological activity of the mature insulin molecule and this requires minimally the introduction of 4 basic amino acids, 2 at either end, i.e., usually Arg-Arg at the B-chain junction and Lys-Arg at the A-chain attachment site. To preserve overall charge neutrality in the molecule then requires the presence of 4 or 5 acidic residues within the C-peptide and these are indeed usually present (Figure 1). As a result, the isoelectric points of proinsulin and insulin are closely similar (~5.3). Interestingly, deletions or alanine replacements of the acidic residues at positions 1, 3, and 4 of the human C-peptide impaired proinsulin folding in vitro (Chen et al., 2002).

During folding, the C-peptide should be flexible (the central glycine-rich region ensures this) and must not interfere with the B or A chains during their folding interactions (evidence suggests that all or most of the folding information is intrinsic to the B and A chains themselves). A factor that may contribute to the "inertness" of the C-peptide is the absence of any aromatic residues (Tyr, Phe, or Trp). However, 4 tyrosines and 3 phenylalanines are present in the insulin molecule and it is likely that C-peptide contacts with these must be avoided during folding (Weiss et al., 1990). There is no space available for the C-peptide in the compactly folded insulin structure and, thus, after folding, it remains outside the insulin moiety as a large unstructured loop. It also does not prevent the self association of proinsulin into dimers and zinc-stabilized hexamers (Blundell et al., 1972; Brems et al., 1990). Indeed, the 6 largely unordered C-peptide loops remain "outside" the spherical structure formed by the 6 densely packed insulin moieties in proinsulin hexamers, somewhat resembling the serpents on a mythical Medusa's head, and serving to block the premature packing of proinsulin hexamers into the crystalline insulin arrays that make up the typical dense granule inclusions as these form during secretory granule maturation (Steiner, 1973; Michael et al., 1987).

**TABLE 1**
Structural constraints on the C-peptide

- Length highly conserved—typically 31 residues (range 25–38)
- Maintains conservation of electrical charge in proinsulin (pI ~5.3)
- Promotes insulin assembly, but does not interact with B or A chain domains
- Does not interfere with transport or sorting to secretory granules
- Enhances interaction with convertases PC1/PC3 and PC2 for efficient conversion to insulin
- Must be stable and compatible with other secretory granule constituents, e.g., IAPP/amylin, others
- Must satisfy structural requirements for acquired physiologic functions

The C-peptide must also support the sorting of proinsulin into the dense core granules as these are formed and it should not interfere with the processing or storage of any of the other granule components. The C-peptide may in fact play a role in stabilizing secretory granules, as it is retained in the soluble phase in the granules where it may interact with and/or stabilize other secretory products such as islet amyloid polypeptide (IAPP or amylin). It has been reported to retard aggregation of human IAPP into amyloid fibrils in vitro (Westermark et al., 1996). Because there are a large number of soluble molecules, including peptides, nucleotides, amino acids, and ions, within

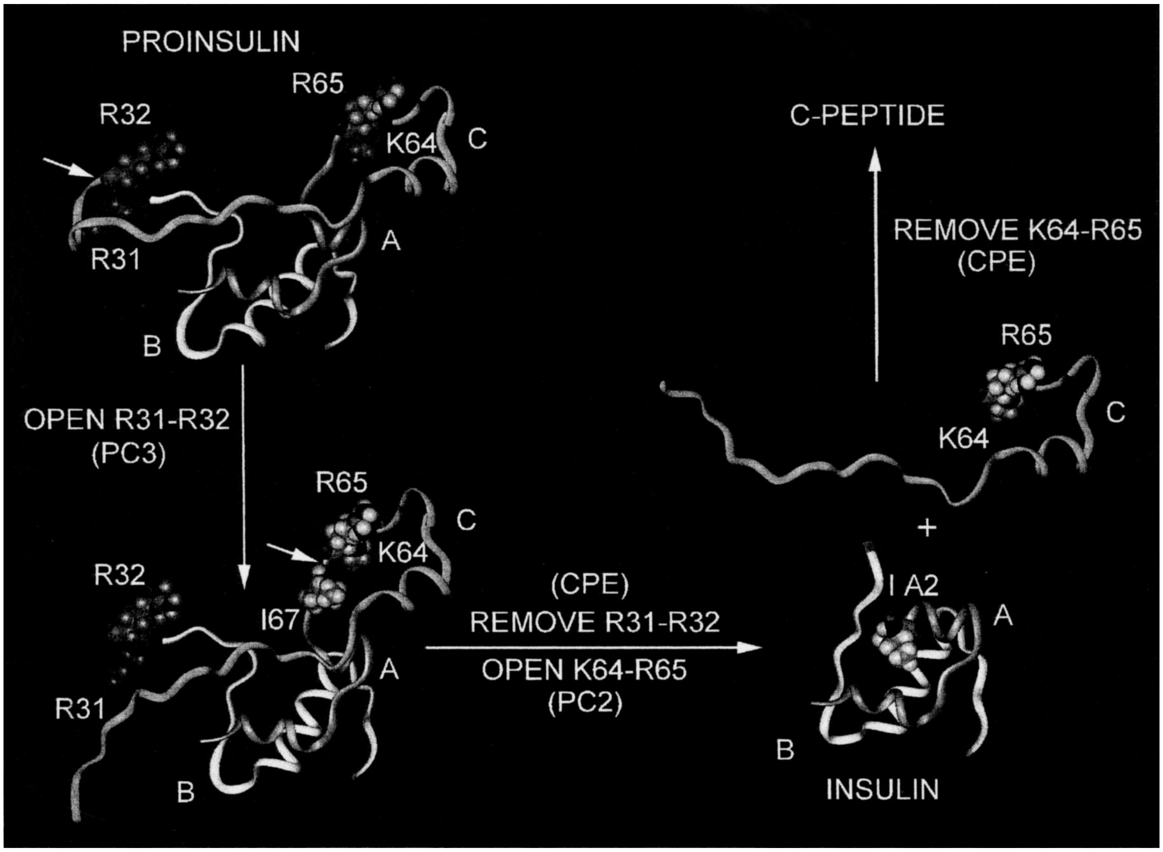

**FIGURE 2**
Normal two-step pathway for the conversion of proinsulin to insulin, based on conformational modeling of the optimal orientations of the cleavage sites (*space-filling residues*) for interaction with the prohormone convertases during proinsulin processing (*upper left panel*). Note the extended C-peptide configuration (*blue ribbon*), which permits the wide separation of the C-end of the B chain (*yellow ribbon*) from the *N*-end of the A chain (*magenta ribbon*), allowing cleavage first after R32 by PC1/3 (*arrow, upper left panel*) and then after R65 by PC2 (*arrow, lower left panel*). To allow cleavage of the C-peptide–A chain junction, the *N*-terminal helix of the A chain must partly unwind to allow I67 to occupy the S2′ subsite of PC2, as indicated in the left panels. After cleavage, I67 resumes its normal position in the hydrophobic core of insulin now designated I A2 (*lower right panel*) (R = arginine; I = isoleucine). (For further modeling details, see Lipkind, 1999.)

the secretory granules, the C-peptide clearly must maintain compatibility with this environment.

Finally, and perhaps most importantly, the C-peptide must facilitate its own excision from proinsulin during the maturation to insulin. Modeling studies have indicated that the length and flexibility of the C-peptide also contributes importantly in this regard as well (Figure 2). At least 8 or 9 residues (6 upstream and 2 or 3 downstream) at the 2 proinsulin processing sites must fit into the catalytic grooves on the convertases as extended $\beta$ strands in order for cleavage to occur (Lipkind and Steiner, 1999). This is more easily accomplished at the B-chain junction, which is highly flexible and can easily assume such a configuration for interaction with PC1/3, assisted by the considerable length and flexibility of the C-peptide and the flexible C-terminal B-chain segment, which is not integral to the folded insulin structure. However, this is more difficult at the A-chain junction where residues $A_1$–$A_3$ are part of an N-terminal alpha helix and the $A_2$-isoleucine side chain is in close contact with the tyrosine side chain of $A_{19}$ within the hydrophobic core of the insulin moiety (Weiss et al., 1990). Hence, this helix must partially unwind, allowing the $A_2$ isoleucine to move out of the insulin core and occupy the S2′ site of the catalytic groove of PC2 (Lipkind and Steiner, 1999). The extended C-peptide, especially after being freed from its B-chain attachment by prior cleavage of this site by PC1/3 (Zhu et al., 2002), may be able to exert sufficient traction on the A-chain helix to assist this structural transition, which allows PC2 to complete the conversion process. After cleavage and dissociation from PC2, the insulin A-chain helix will spontaneously reform, allowing the $A_2$ isoleucine to reestablish its internal contact within the insulin molecule. Thus, here again, in this critical process, we see that the C-peptide length plays both an active as well as a passive role.

### BIOLOGICAL ACTIONS OF C-PEPTIDE

Surely the very high degree of conservation of an overall length in the C-peptide of about 30 residues, as well as the other structural considerations discussed above, provide an adequate functional framework to account for the degree of evolutionary conservation seen in the C-peptide. This region of proinsulin accepts mutations at a rate that is 12 to 15 times faster than the rate of evolutionary change in insulin, which is constrained not only by its own receptor-binding requirements, but those of maintaining a clear-cut duality of recognition to differentiate it from its sibling molecules, especially the closely related IGFs. On this basis, it is tempting to conclude that were the C-peptide additionally constrained by requirements for interacting with a receptor of its own, we might see some higher degree of conservation of structure somewhere within the C-peptides, but this does not appear to be the case, as the variability extends throughout the molecule. Nonetheless, a number of physiological functions of the C-peptide, ranging from stimulation of sodium-potassium ATPase activity, altered neural and vascular function, to even some insulin-like actions, have been described in recent years (Wahren et al., 1994; Kitamura et al., 2001; Ido et al., 1997) or are discussed in articles included in this volume. A putative C-peptide receptor has also been identified, but has not yet been isolated or characterized (Rigler et al., 1999; Henriksson et al., 2001). It will be of great interest to learn more about this receptor molecule and its recognition properties. It is unlikely that C-peptide interacts directly with lipid membranes (Henriksson et al., 2000). However, it should be borne in mind that peptides can be taken up by cells via endocytosis or possibly also pinocytosis, and could exert actions within cells that might not require conservation of structure beyond that seen in the mammalian C-peptides. It certainly seems likely that the C-peptide may be a structure that has acquired additional physiological functions during mammalian evolution. Indeed, the C-peptide is a fascinating entity, beginning with its original vital role in ensuring the synthesis and correct assembly of the insulin molecule, but perhaps also going beyond that role to assume further important endocrine functions of its own by virtue of its temporal association with insulin in the ebb and flow of metabolism over the eons.

### REFERENCES

Blundell, T., Dodson, G., Hodgkin, D., et al. (1972) Insulin: The structure in the crystals and its reflection in chemistry and biology. *Adv. Protein Chem.,* **26,** 279.

Brems, D. N., Brown, P. L., Heckenlaible, L. A., and Frank, B. H. (1990) Equilibrium denaturation of insulin and proinsulin. *Biochemistry,* **29,** 9289–9293.

Brogiolo, W., Stocker, H., Ikeya, T., Rintelen, F., Fernandez, R., and Hafen, E. (2001) An evolutionarily conserved function of the *Drosophila* insulin receptor and insulin-like peptides in growth control. *Curr. Biol.,* **11,** 213–221.

Burkhardt, E., Adham, I. M., Hobohm, U., Murphy, D., Sander, C., and Engel, W. (1994) A human cDNA coding for the Leydig insulin-like peptide (Ley I-L). *Hum. Genet.,* **94,** 91–94.

Cara, J. F., Mirmira, R. G., Nakagawa, S. H., and Tager, H. S. (1990) An insulin-like growth factor I/insulin hybrid exhibiting high potency for interaction with the type I insulin-like growth factor and insulin receptors of placental plasma membranes. *J. Biol. Chem.,* **265,** 17820–17825.

Chan, S., Cao, Q.-P., and Steiner, D. (1990) Evolution of the insulin superfamily: Cloning of a hybrid insulin/insulin-like growth factor cDNA from amphioxus. *Proc. Natl. Acad. Sci. U. S. A.,* **87,** 9319–9323.

Chen, L. M., Yang, X. W., and Tang, J. G. (2002) Acidic residues on the N-terminus of proinsulin C-peptide are important for the folding of insulin precursor. *J. Biochem. (Tokyo),* **131,** 855–859.

Day, R., Lazure, C., Basak, A., Boudreault, A., Limperis, P., Dong, W., and Lindberg, I. (1998) Prodynorphin processing by proprotein convertase 2. Cleavage at single basic residues and enhanced processing in the presence of carboxypeptidase activity. *J. Biol. Chem.,* **273,** 829–836.

De Meyts, P., and Whittaker, J. (2002) Structural biology of insulin and IGF1 receptors: Implications for drug design. *Nat. Rev. Drug Discov.,* **1,** 769–783.

Derewenda, U., Derewenda, Z., Dodson, E., Dodson, G., Bing, X., and Markussen, J. (1991) X-ray analysis of the single chain B29-A1 peptide-linked insulin molecule. A completely inactive analogue. *J. Mol. Biol.,* **220,** 425–433.

Duguay, S., Milewski, W., Young, B., Nakayama, K., and Steiner, D. (1997) Processing of wild-type and mutant proinsulin-like growth factor-IA by subtilisin-related proprotein convertases. *J. Biol. Chem.,* **272,** 6663–6670.

Duguay, S. J., Jin, Y., Stein, J., Duguay, A. N., Gardner, P., and Steiner, D. F. (1998) Post-translational processing of the insulin-like growth factor-2 precursor. Analysis of *O*-glycosylation and endoproteolysis. *J. Biol. Chem.,* **273,** 18443–18451.

Duret, L., Guex, N., Peitsch, M. C., and Bairoch, A. (1998) New insulin-like proteins with atypical disulfide bond pattern characterized in *Caenorhabditis elegans* by comparative sequence analysis and homology modeling. *Genome Res.,* **8,** 348–353.

Fricker, L., Evans, C., Esch, F., and Herbert, E. (1986) Cloning and sequence analysis of cDNA for bovine carboxypeptidase E. *Nature,* **323,** 461.

Furuta, M., Carroll, R., Martin, S., Swift, H., Ravazzola, M., Orci, L., and Steiner, D. (1998) Incomplete processing of proinsulin to insulin accompanied by elevation of des-31,32 proinsulin intermediate in islets of mice lacking active PC2. *J. Biol. Chem.,* **273,** 3431–3437.

Henriksson, M., Pramanik, A., Shafqat, J., Zhong, Z., Tally, M., Ekberg, K., Wahren, J., Rigler, R., Johansson, J., and Jornvall, H. (2001) Specific binding of proinsulin C-peptide to intact and to detergent-solubilized human skin fibroblasts. *Biochem. Biophys. Res. Commun.,* **280,** 423–427.

Henriksson, M., Shafqat, J., Liepinsh, E., Tally, M., Wahren, J., Jörnvall, H., and Johansson, J. (2000) Unordered structure of proinsulin C-peptide in aqueous solution and in the presence of lipid vesicles. *Cell. Mol. Life. Sci.,* **57,** 337–342.

Hua, Q. X., Jia, W., Frank, B. H., Phillips, N. F., and Weiss, M. A. (2002) A protein caught in a kinetic trap: Structures and stabilities of insulin disulfide isomers. *Biochemistry,* **41,** 14700–14715.

Hudson, P., John, M., Crawford, R., Haralambidis, J., Scanlon, D., Gorman, J., Tregear, G., Shine, J., and Niall, H. (1984) Relaxin gene expression in human ovaries and the predicted structure of a human preprorelaxin by analysis of cDNA clones. *EMBO J.,* **3,** 2333–2339.

Ido, Y., Vindigni, A., Chang, K., Stramm, L., Chance, R., Heath, W. F., DiMarchi, R. D., Di Cera, E., and Williamson, J. R. (1997) Prevention of vascular and neural dysfunction in diabetic rats by C-peptide. *Science,* **277,** 563–566.

Kitamura, T., Kimura, K., Jung, B. D., Makondo, K., Okamoto, S., Canas, X., Sakane, N., Yoshida, T., and Saito, M. (2001) Proinsulin C-peptide rapidly stimulates mitogen-activated protein kinases in Swiss 3T3 fibroblasts: requirement of protein kinase C, phosphoinositide 3-kinase and pertussis toxin-sensitive G-protein. *Biochem. J.,* **355,** 123–129.

Koman, A., Cazaubon, S., Couraud, P.-O., Ulrich, A., and Strosberg, A. D. (1996) Molecular characterization and in vitro biological activity of placentin, a new member of the insulin gene family. *J. Biol. Chem.,* **271,** 20238–20241.

Kondo, H., Ino, M., Suzuki, A., Ishizaki, H., and Iwami, M. (1996) Multiple gene copies for bombyxin, an insulin-related peptide of the silkmoth *Bombyx mori*: Structural signs for gene rearrangement and duplication responsible for generation of multiple molecular forms of bombyxin. *J. Mol. Biol.,* **259,** 926–937.

Kwok, S., Chan, S., and Steiner, D. (1983) Cloning and nucleotide sequence analysis of the dog insulin gene: Coded amino acid sequence of canine preproinsulin predicts an additional C-peptide fragment. *J. Biol. Chem.,* **258,** 2357–2363.

Lagueux, M., Lwoff, L., Meister, M., Goltzene, F., and Hoffmann, J. (1990) cDNAs form neurosecretory cells of brains of locusta migratoria (insecta, orthoptera) encoding a novel member of the superfamily of insulins. *Eur. J. Biochem.,* **187,** 249–254.

LeRoith, D., and Roberts, C. T., Jr. (1993) Insulin-like growth factors. *Ann. N. Y. Acad. Sci.,* **692,** 1–9.

Lipkind, G., and Steiner, D. (1999) Predicted structural alterations in proinsulin during its interactions with prohormone convertases. *Biochemistry,* **38,** 890–896.

Liu, M., Ramos-Castaneda, J., and Arvan, P. (2003) Role of the connecting peptide in insulin biosynthesis. *J. Biol. Chem.,* **278,** 14798–14805.

Michael, J., Carroll, R., Swift, H. H., and Steiner, D. F. (1987) Studies on the molecular organization of rat insulin secretory granules. *J. Biol. Chem.,* **262,** 16531–16535.

Muller, L., and Lindberg, I. (1999) The cell biology of the prohormone convertases PC1 and PC2. In: *Progress in Nucleic Acids Research*, Vol. 63, Edited by Moldave, L., pp. 69–108. San Diego, Academic Press.

Naggert, J., Fricker, L., Varlamov, O., Nishina, P., Rouille, Y., Steiner, D., Carroll, R., Paigen, B., and Leiter, E. (1995) Hyperproinsulinemia in obese fat/fat mice is associated with a point mutation in the carboxypeptidase E gene and reduced carboxypeptidase activity in the pancreatic islets. *Nat. Genet.,* **10,** 135–142.

Nishi, M., Sanke, T., Nagamatsu, S., Bell, G., and Steiner, D. (1990) Islet amyloid polypeptide. A new beta cell secretory product related to islet amyloid deposits. *J. Biol. Chem.,* **265,** 4173–4176.

Nolan, C., Margoliash, E., Peterson, J., and Steiner, D. (1971) The structure of bovine proinsulin. *J. Biol. Chem.,* **246,** 2780–2795.

Peterson, J., Nehrlich, S., Oyer, P., and Steiner, D. (1972) Determination of the amino acid sequence of the monkey, sheep, and dog proinsulin C-peptides by a semi-micro Edmon degradation procedure. *J. Biol. Chem.,* **247,** 4866–4871.

Polonsky, K. S., Licinio-Paixao, J., Given, B. D., Pugh, W., Rue, P., Galloway, J., Karrison, T., and Frank, B. (1986) Use of biosynthetic human C-peptide in the measurement of insulin secretion rates in normal volunteers and type I diabetic patients. *J. Clin. Invest.,* **77,** 98–105.

Polonsky, K. S., and O'Meara, N. M. (2001) Secretion and metabolism of insulin, proinsulin, and C-peptide. In: *Endocrinology,* Vol. 1, Edited by DeGroot, L. J., and Jameson, J. L., pp. 697–711. Philadelphia, W. B. Saunders.

Powell, S. K., Orci, L., Craik, C. S., and Moore, H.-P. H. (1988) Efficient targeting to storage granules of human proinsulins with altered propeptide domain. *J. Cell. Biol.,* **106,** 1843–1851.

Rhodes, C. J., and Alarcón, C. (1994) What $\beta$-cell defect could lead to hyperproinsulinemia in NIDDM? Some clues from recent advances made in understanding the proinsulin-processing mechanism. *Diabetes,* **43,** 511–517.

Rigler, R., Pramanik, A., Jonasson, P., Kratz, G., Jansson, O. T., Nygren, P.-A., Stahl, S., Ekberg, K., Johansson, B.-L., Uhlen, S., Uhlen, M., Jornvall, H., and Wahren, J. (1999) Specific binding of proinsulin C-peptide to human cell membranes. *Proc. Natl. Acad. Sci.,* **96,** 13318–13323.

Rubenstein, A., Clark, J., Melani, F., and Steiner, D. (1969) Secretion of proinsulin C-peptide by pancreatic B cells and its circulation in blood. *Nature,* **224,** 697–699.

Seidah, N. G., and Chrétien, M. (1999) Proprotein and prohormone convertases: A family of subtilases generating diverse bioactive polypeptides. *Brain Res.,* **848,** 45–62.

Smit, A. B., Spijker, S., Van Minnen, J., Burke, J. F., De Winter, F., Van Elk, R., and Geraerts, W. P. (1996) Expression and characterization of molluscan insulin-related peptide VII from the mollusc Lymnaea stagnalis. *Neuroscience,* **70,** 589–596.

Steiner, D. (1973) Cocrystallization of proinsulin and insulin. *Nature,* **243,** 528–530.

Steiner, D., Bell, G., Rubenstein, A., and Chan, S. (2000a) Chemistry and biochemistry of pancreatic protein hormones. In: *Endocrinology*, Edited by DeGroot, L., and Jameson, J., pp. 667–696. Philadelphia, W. B. Saunders.

Steiner, D., Chan, S., and Rubenstein, A. (2000b) Biosynthesis of insulin. In: *Handbook of Physiology, The Endocrine System*, Vol. II, Edited by Jefferson, L., and Cherrington, A., pp. 49–77. New York, Oxford University Press.

Steiner, D. F. (1984) The biosynthesis of insulin: Genetic, evolutionary and pathophysiologic aspects. In: *The Harvey Lectures, Series 78*, Edited by Gotshlich, E. C., pp. 191–228. New York, Academic Press.

Steiner, D. F., Clark, J. L., Nolan, C., Rubenstein, A. H., Margoliash, E., Aten, B., and Oyer, P. (1969) Proinsulin and the biosynthesis of insulin. *Rec. Prog. Horm. Res.,* **25,** 207–292.

Verchere, C., Paoletta, M., Neerman-Arbez, M., Rose, K., Irminger, J., Gingerich, R., Kahn, S., and Halban, P. (1996) Des-(27–31) C-peptide. A novel secretory product of the rat pancreatic beta cell produced by truncation of proinsulin connecting peptide in secretory granules. *J. Biol. Chem.,* **271,** 27475–27481.

Wahren, J., Johansson, B.-L., and Wallberg-Henriksson, H. (1994) Does C-peptide have a physiological role? *Diabetologia,* **37,** S99–S107.

Wei, G., Hu, M. H., and Tang, J. G. (1995) Double-C-peptide human proinsulin. *Biochem. Mol. Biol. Int.,* **35,** 37–46.

Weiss, M. A., Frank, B., Khait, I., Pekar, A., Heiney, R., Shoelson, S., and Neuringer, L. (1990) NMR and photo-CIDNP studies of human proinsulin and prohormone processing intermediates with application to endopeptidase recognition. *Biochemistry,* **29,** 8389–8401.

Westermark, P., Li, Z. C., Westermark, G. T., Leckstrom, A., and Steiner, D. F. (1996) Effects of beta cell granule components on human islet amyloid polypeptide fibril formation. *FEBS Lett.,* **379,** 203–206.

Zhou, A., Webb, G., Zhu, X., and Steiner, D. F. (1999) Proteolytic processing in the secretory pathway. *J. Biol. Chem.,* **274,** 20745–20748.

Zhu, X., Orci, L., Carroll, R., Norrbom, C., Ravazzola, M., and Steiner, D. F. (2002) Severe block in processing of proinsulin to insulin accompanied by elevation of des-64,65 proinsulin intermediates in islets of mice lacking prohormone convertase 1/3. *Proc. Natl. Acad. Sci. U. S. A.,* **99,** 10299–10304.

